# Towards unraveling antimicrobial resistance dynamics: a longitudinal exploration of rectal swab metagenomes

**DOI:** 10.1186/s12866-025-03874-z

**Published:** 2025-03-17

**Authors:** Sébastien Boutin, Nadja Käding, Meriem Belheouane, Matthias Merker, Jan Rupp, Dennis Nurjadi

**Affiliations:** 1 Institute of Medical Microbiology, University of Lübeck and University Hospital Schleswig-Holstein Campus Lübeck, Lübeck, Germany; 2 German Center for Infection Research (DZIF), Partner Site Hamburg-Lübeck-Borstel-Riems, Lübeck, Borstel Germany; 3 Airway Research Center North (ARCN), German Center for Lung Research (DZL), Lübeck, Germany; 4 Infectious Diseases Clinic, University Hospital Schleswig-Holstein, Campus Lübeck, Lübeck, Germany; 5 Evolution of the Resistome, Research Center Borstel, Borstel, Germany

**Keywords:** Clinical metagenomic, Antimicrobial resistance, MDRO colonisation, Rectal swabs

## Abstract

The increasing prevalence of antimicrobial resistance (AMR) poses significant challenges in clinical settings. In particular, early screening and detection of colonization by multidrug-resistant organisms (MDROs) in patients at admission is crucial. In this context, the clinical use of metagenomics (mNGS) holds promise for fast and untargeted diagnostic methods. Here, we aimed to evaluate the long-term stability of the rectal microbiome and the diagnostic accuracy of mNGS in comparison to culture and whole-genome sequencing (WGS) of MDROs. We analyzed rectal swabs from 26 patients with two consecutive admissions over a four-year period. The detected antimicrobial resistance genes and assembled metagenomes were compared to those obtained via classical culture-based antimicrobial susceptibility testing and WGS of isolated MDROs. Our results showed that the rectal microbiome is variable during the two timepoints, highlighting the variability in the niche. Nevertheless, we also observed strong co-occurrence of taxa, suggesting that the rectal swab microbiome is also a regulated environment with cooperative biotic interactions. In total, we isolated and sequenced 6 MDROs from 6 patients at individual timepoints. Almost all AMR genes from the genomes of the isolates (median: 100%, range: 84.6–100%) could be detected by mNGS of the rectal swabs at the time of isolation of the MDRO but not at the time of culture negativity. In addition, we detected AMR genes and potentially pathogenic species in patients with negative cultures. In conclusion, our study showed that, in principle, mNGS of rectal swabs can detect clinically relevant AMR profiles. However, the cooccurrence of AMR genes and potentially-pathogenic species does not always correlate with culture-based diagnostic results but rather indicates a potential risk of horizontal AMR gene transfer. However, it is unclear whether the observed discrepancies are due to transient or locally confined colonization of MDROs, limits of detection, or variability of the sampling method and specimens.

**Supplementary Information:**

The online version contains supplementary material available at 10.1186/s12866-025-03874-z.

## Background

The acquisition and increasing rate of antimicrobial resistance (AMR) due to antibiotic exposure are major concerns, especially in clinical settings where the treatment of multidrug-resistant organisms (MDROs) is difficult [1]. The two most common mechanisms for acquiring resistance are the development of mutations that alter target genes or the acquisition of mobile genetic elements (MGEs) carrying antimicrobial resistance genes (ARGs). Apart from transmission events between patients, healthcare workers and the hospital environment, the patient microbiome should be considered a potential reservoir of ARG-containing MGEs.

In hospitalized patients, MDROs can be acquired both in the hospital and the community prior to hospitalization. The latter is a major challenge because asymptomatic MDRO carriage may be missed by infection prevention measures. Furthermore, community-acquired MDROs are also a major concern, as patients may bring MDROs to hospitals and transmit them to other patients. Studies have shown that once acquired, MDRO colonization can persist in the gut microbiome for almost 2 years [2]. In this context, asymptomatic colonization with MDROs such as vancomycin-resistant enterococci (VREs) and third-generation cephalosporin-resistant Enterobacterales (3GCREB) often goes undetected due to local infection prevention and control policies to only screen for MDRO carriage in high-risk patients, such as immunocompromised hematology/oncology patients [3]. Although traditional diagnostic methods, including selective culture and targeted molecular approaches, have proven effective in the past, the development of high-throughput sequencing and metagenomics (mNGS) holds promise for untargeted diagnostic methods [4, 5].

mNGS involves sequencing whole DNA from clinical samples to identify microorganisms and antimicrobial resistance determinants. As an unbiased approach, it expands the range of pathogens detected and offers the potential to detect the genomic content and, therefore, predict antimicrobial resistance to guide tailored treatment. It has been applied to various types of specimens, and despite its potential to rapidly identify a wide range of pathogens, the cost-effectiveness, complexity of microbial ecology and practical implications in clinical settings are not yet fully understood [6].

Rectal swabbing has been established as the most applicable sampling method to screen for MDRO colonization on admission. Extensive studies have been performed comparing rectal swabs and stool samples, showing discrepant results, either validating or invalidating rectal swabs as good surrogates or proxies for the study of gut microbes [7–10]. However, swabs have advantages over stool because they are always available, easy to collect and more compliant than stool or colonoscopic sampling. It is, therefore, the sample of choice for admission screening and detection of pathogens and AMR at admission, but studies demonstrating the reliability of mNGS compared to classical culture and the stability of the rectal microbiome are sparse and/or often cross-sectional.

In this proof-of-concept observational study, we aimed to understand whether rectal swabs can be used for AMR profiling using mNGS and whether ARGs can be detected in culture-negative samples collected before and/or after culture positivity. We longitudinally analyzed patients who were admitted to 2 consecutive hospitals over a 4-year period to gain insight into the dynamics of the rectal microbiome, especially the resistome, and the efficiency of mNGS of rectal swabs for detecting AMR genes and pathogens. We also compared the AMR profiles of the resistome and isolated MDROs to evaluate the performance of mNGS in detecting resistance determinants.

## Methods

### Study setting and study population

This study analyzed a subset of datasets from a large German multicenter cohort of six tertiary care centers (R-Net consortium, 2016–2019), which included 500 patients per year per center, to investigate the prevalence of colonization by multidrug-resistant bacteria (3rd generation cephalosporin, carbapenem-resistant Enterobacteriaceae or vancomycin-resistant enterococci) using rectal swabs at admission (from day 1 to 3). The inclusion criterion was 500 patients per year per center from stationary patients older than 18 years, excluding patients from ophthalmology, pediatry, psychiatry or intensive care units. From one participating center, 30 patients who met the inclusion criteria were identified because they were screened on at least two separate occasions at admission within the 4 years of the study. Four of the 30 patients were excluded because of nonrecoverable samples from one of the time points or insufficient DNA quantity for metagenomic sequencing. The cohort was balanced regarding sex (42% male), and the average age was 69 years (range 54–81). The samples were collected using a Copan eSwab with Amies media (80490CEA). To obtain the intrarectal swab, the swabs was inserted 5 cm deep into the rectum and then rotated 3x to gain enough material. The swab remains still several seconds to ensure better material absorption. The swab is then withdrawn and placed into the eSwab transport tube for culture.

### Bacterial identification and susceptibility testing

The media from the rectal swabs tube were incubated using a 10 µL loop and following the 3-phase streaking on ESBL selective agar (chromID ESBL, bioMérieux), CRE selective agar (MAST CHROMAgar, mSuperCARBA, Mast Diagnostica GmbH), VRE selective agar (chromID VRE, bioMérieux) and MacConkey agar for growth control at 35 ± 1 °C for 24–48 h. From all the chromogenic selective agar plates with positive findings (enterobacteria and enterococci), species were determined by mass spectrometry (MALDI-TOF) or biochemical analysis (VITEK 2) and subjected to antibiotic susceptibility testing (VITEK 2). The isolates were also subjected to whole-genome sequencing. Bacterial DNA was isolated from overnight cultures using the PureLink Genomic DNA Isolation Kit (Thermo Fisher Scientific, Germany), and libraries were prepared using the Nextera XT Kit (Illumina). Individually tagged libraries were sequenced (2 × 300 bp/2 × 150 bp) on the MiSeq/NextSeq platform (Illumina). The remaining liquid from the rectal swabs were frozen at -80 °C until DNA extraction.

### DNA extraction from rectal swabs

The rectal swab samples were thawed at 4 °C overnight. After that, the samples were subjected to high-speed vortexing for 1 min, and then the solution (the preservation buffer in which the cotton bud was immersed) was transferred to a clean 2 ml tube and centrifuged at high speed (10,000 g) for 15 min at 4 °C. The supernatant was discarded, and the pellet was retained. An equal volume of PCR-clean water was added to the resulting cell debris/pellet and incubated at RT for 5 min to osmotically lyse the mammalian cells as described by Marotz et al., 2018 [11]. Subsequently, the samples were subjected to DNase treatment with 1 µl (3 units) Turbo Dnase for each sample (Dnase Kit Turbo Dnase, Ambion) and incubated at 37 °C for 30 min. After incubation, 50 µl of 1X PBS was added, and the samples were placed on ice. To each sample, 300 µl of power bead solution, 50 µl of SL buffer (DNeasy UltraClean Microbial Kit, Qiagen), and 20 µl of proteinase K (20 mg/ml) were added. In addition, extraction negative blank samples were processed along with the rectal samples. All samples (rectal and negative controls) were placed on a Fast-Prep machine (MP Biomedicals), homogenized at a speed of 6.5 M/S for 15 s three times, and then centrifuged at 10,000 × g for 2 min, after which the supernatant was transferred to a clean 2 ml tube. After the homogenization step, DNA extraction was carried out following the manufacturer´s protocol (DNeasy UltraClean Microbial Kit, Qiagen), and the DNA was eluted in 50 µl of Solution EB. All negative controls presented a DNA concentration below the minimum threshold (< 0.05 ng/µL). A 16 S rRNA PCR (V3-V4 region) was performed on these negative controls and showed no visible amplification on the DNA fragment analyzer.

### Whole-metagenomic sequencing

DNA libraries with an average size of 700 bp were prepared as described previously [12] using the Nextera XT library kit (Illumina, San Diego, CA, USA). Quality and quantity controls of the extracted DNA and DNA libraries were performed with a Qubit (Thermo Fisher, USA) and a fragment analyzer system (Agilent Technologies, CA, USA). Sequencing was performed on an Illumina NextSeq 2000 with 2 × 150 bp P3 reagents.

### Bioinformatics

Raw fastq files were processed through quality control using fastp (v0·23·1 with parameters -q = 30 and -l = 45) to remove/trim bad quality sequences and remove the adapters [13]. Metagenomic reads were decontaminated using Kneaddata (https://github.com/bioBakery/kneaddata) with the human genome GRCh38 as a reference for decontamination. Clean reads were then assembled with SPAdes 3.15.5 with the additional option “-meta” for the metagenomic reads [14]. For the isolated bacterial genome, the draft genomes were curated by removing contigs with a length < 500 bp and/or coverage < 10×. The quality of the final draft was quality controlled using Quast (v5·0·2) [15]. The species identification of each draft genome was performed using mash (subcommand screen) [16] by screening each draft genome to a database composed of a representative genome of each species present in the Microbial Genomes resource (https://www.ncbi.nlm.nih.gov/genome/microbes/). The obtained isolate genomes and metagenomes were screened for antimicrobial resistance genes and plasmid type using Abricate (-minid 90 and -mincov 50) (https://github.com/tseemann/abricate with the NCBI, CARD, ARG-ANNOT, ResFinder, MEGARES and plasmidfinder databases) [17–22]. To evaluate the presence of antimicrobial resistance genes, we used an assembly based approach to ensure better coverage of the genes. The coverage minimum was lower than that for the bacterial genome screening due to the fragmented nature of the metagenomic data. The clean metagenomic reads were also processed through Metaphlan 4.0 to profile the taxonomy of the metagenomes to the species levels, and the species of interest were further studied at the strain level using Strainphlan 4.1.0 [23, 24].

### Data analysis

Analysis of the microbiota was done using the taxon’s relative abundance in % from the Metaphlan output and presence/absence of resistance genes was performed using R 4.3.3 and the packages Phyloseq, Complexheatmap and ggplot2. The impact of clinical parameters (age, sex, antibiotic usage) and time on the alpha diversity, resistome and plasmidome was evaluated using a linear mixed-effects model with a random effect for the patient ID.

### Ethical considerations

The study was approved by the ethics committee of the coordinating site (Cologne) and all sampling sites (Ethical Committee Approval, Coordinating Site Cologne: EA4/018/14, Clinical Trial Registration: NCT04937361, registration date: 2021-06-23).

### Funding

This work was supported by the German Centre for Infection Research (DZIF), project numbers TTU 08.811 and TTU 08.824.

## Results

### Longitudinal analysis of the rectal microbiota

**Fig. 1:**
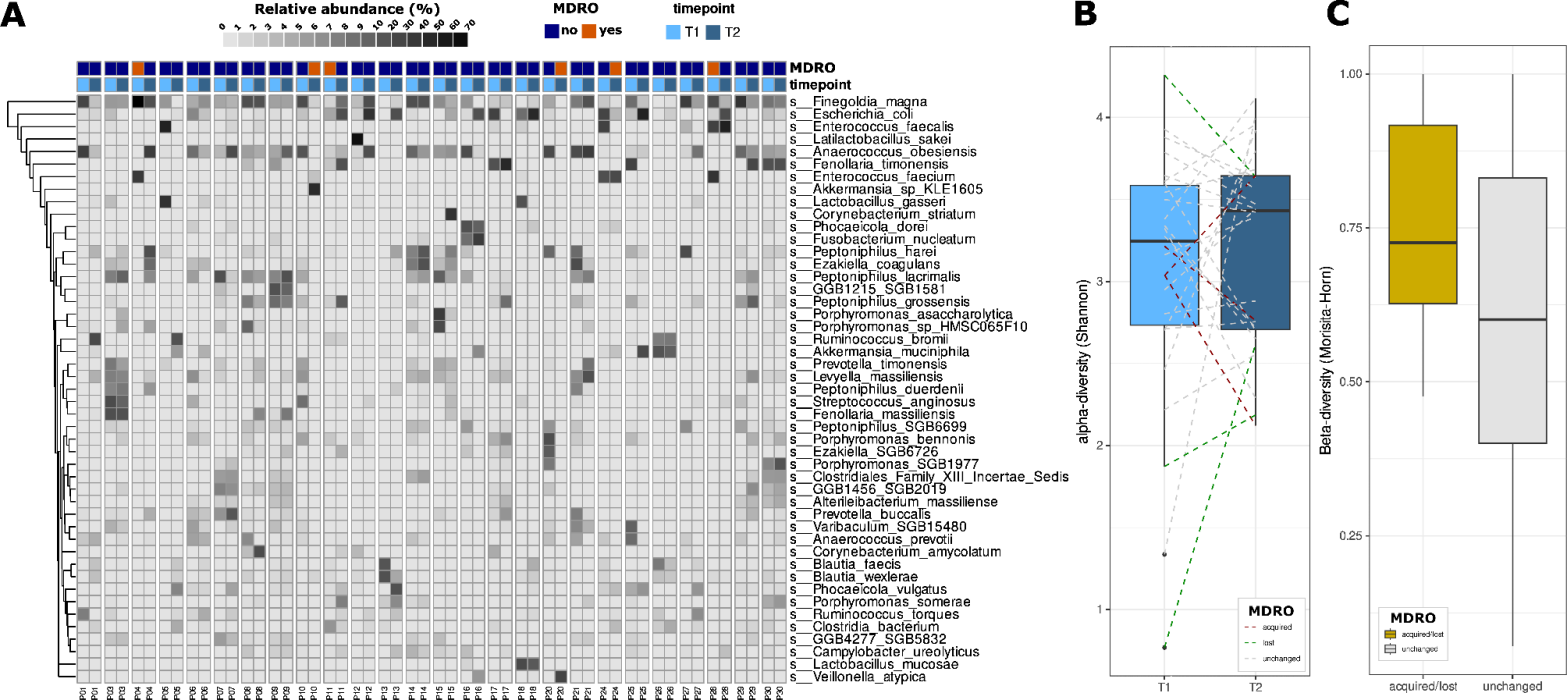
Microbial composition of the rectal swabs in our study. (**A**) Heatmap representing the relative abundance of the 46 most abundant species (mean rel. abundance > 0.5%). The detection by culture of an MDRO is color coded in orange/blue while the timepoint is color-coded in light blue/dark blue shading (**B**) α-diversity (Shannon index) between the two time points of the study. (**C**) β-diversity (Morisita-Horn index) within patients during the acquisition or loss of a culturable MDRO

All samples were sequenced to a depth > 10 Mio non-human paired reads. The average paired read number was 19 Mio (11–24 Mio). In total, 1144 microbial species were found in the rectal swabs. The majority of the microbial species are bacteria, only 2 species belong to the Eukaryota Kingdom (*Candida albicans* and *Candida tropicalis*), and 5 species are Archae, with only 2 members classified to the species level (*Methanobrevibacter smithii* & *Methanosphaera stadtmanae*). Only 52 species had an average abundance greater than 0.5% in the overall population, and the 18 most abundant species (relative abundance > 1%) found in the samples were *Finegoldia magna* (7.72%), *Anaerococcus obesiensis* (5.23%), *Escherichia coli* (5.20%), *Enterococcus faecalis* (2.80%), *Fenollaria timonensis* (2.71%), *Peptoniphilus lacrimalis* (2.39%), *Peptoniphilus harei* (1.95%), *Enterococcus faecium* (1.81%), *Peptoniphilus grossensis* (1.63%), *Levyella massiliensis* (1.60%), *Ezakiella coagulans* (1.28%), *Latilactobacillus sakei* (1.15%), *Porphyromonas bennonis* (1.13%), *Akkermansia muciniphila* (1.09%), *Lactobacillus gasseri* (1.05%), *Prevotella buccalis* (1.05%), *Phocaeicola vulgatus* (1.02%), and *Anaerococcus prevotii* (1.00%) (Fig. 1A). Co-occurrence analysis revealed that 28 species co-occurred positively (Rho > 0.5 and p-value < 0.01), and *Akkermansia muciniphila* was negatively correlated with the relative abundances of *Finegoldia magna*, *Anaerococcus obesiensis and Peptoniphilus lacrimalis* (Supplementary Fig. 1). We did not observe a significant change in the α-diversity (Shannon index) between either the two time points (ANOVA with linear mixed effect model with patient ID as a random effect: p-value = 0.38, Fig. 1B) or the carriage of an MDRO (Wilcoxon test, p-value = 0.27). We observed a high β-diversity (Morisita-Horn index) within patients between the time points, and the acquisition or loss of an MDRO seemed to increase the β-diversity without reaching significance (Wilcoxon test, p-value = 0.22). We also observed a slight impact of the time difference between the time points on the β-diversity, but the difference did not reach significance (Supplementary Fig. 2).

### Culture of MDROs

From the 26 patients included, 6 MDROs from 6 patients were isolated four 3GCREB strains (*Escherichia coli* (*n* = 2, P11 & P28), *Klebsiella aerogenes* (*n* = 1, P20), and *Klebsiella pneumoniae* (*n* = 1, P24)) and two vancomycin-resistant *Enterococcus faecium strains* (P04 & P10). Three patients acquired an MDRO during the study period (P10; 2-year interval, P20 and P24; 1-year interval), and three patients lost the MDRO during the study period (P11; 2-year interval, P04 and P28; 1-year interval). None of the patients were colonized at the two sampling time points.

### Longitudinal analysis of the resistome

**Fig. 2:**
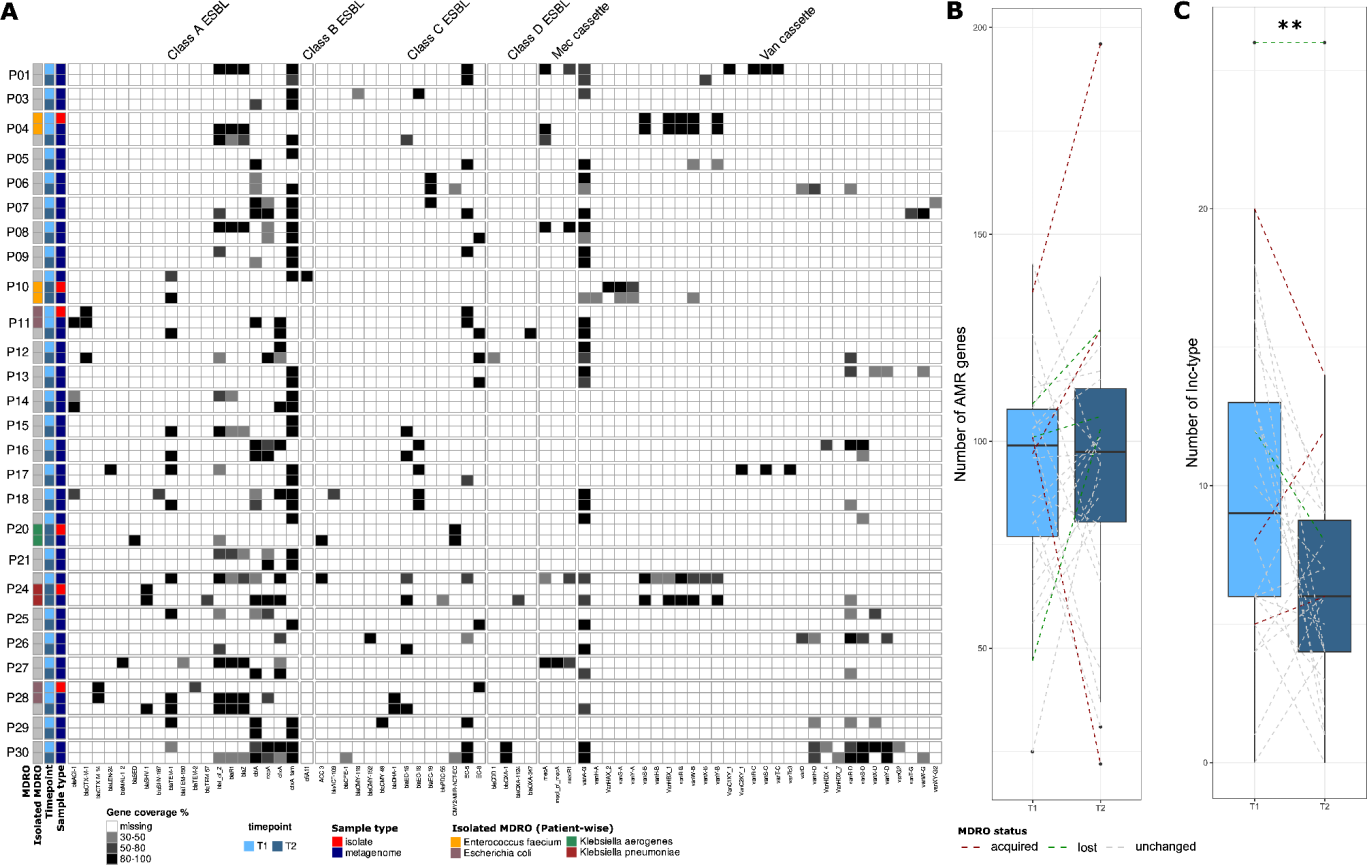
Comparative analysis of the AMR genes in rectal swabs metagenomes and the isolated MDROs. (**A**) Heatmap of the detection of beta-lactamase genes, Van and mec cassettes in pairwise comparisons of the metagenomes and the isolated genomes. The column isolated MDRO indicate the timepoint and species of MDRO isolated. (**B**) Number of antimicrobial resistance genes and (**C**) typeable plasmid between the two time points of the study

In total, 71 genes associated with resistance to cephalosporin, methicillin or vancomycin were found in the population, and most of them were prevalent in samples where no MDRO was isolated. The most prevalent beta-lactamase genes are associated with Bacteroides species such as *cfxA*-like and *cfxA* (*n* = 32, 55%; *n* = 14, 24%), *cblA* (*n* = 20, 34%) and *cepA* (*n* = 14, 24%), as well as genes involved in beta-lactamase PC1 (*blaZ*) expression (*blaZ*: *n* = 11, 19%; *blaR*: *n* = 12, 21%; *blaI*: *n* = 19, 33%), which are often associated with Gram-positive bacteria such as *Bacillus*, *Enterococcus* and *Staphylococcus* species. The genes from the *bla*_EC_ family associated mostly with *E. coli* and *Shigella* species were also highly prevalent (*bla*_EC−5_: *n* = 13,22%; *bla*_EC−15_: *n* = 7,12%; *bla*_EC−8_: *n* = 6,10%; *bla*_EC−18_: *n* = 5,9%; *bla*_EC−19_: *n* = 3,5%). Finally, several beta-lactamase genes that are transferable among Enterobacteriaceae were also detected (*bla*_TEM−1_: *n* = 13, 22%; *bla*_TEM−150_: *n* = 1, 2%; *bla*_TEM−2_: *n* = 1, 2%; *bla*_TEM−57_: *n* = 1, 2%; *bla*_CTX−M−1_: *n* = 3, 5%; *bla*_CTX−M−14_: *n* = 2, 3%; *bla*_SHV−1_: *n* = 3, 5%; *bla*_SHV−187_: *n* = 1, 2%). The *mec* cassette was found at least partially in 6 samples (10%), the *van* cassette was found in 37 samples (71%), and the operons *vanB* and *vanD* were the most prevalent (Fig. 2A).

Interestingly, the ESBL genes or *van* cassette from the MDROs isolated were found in every corresponding MDRO-carrying metagenome, with a certain limitation for the *van* cassette, which was found only partially in patient 10. However, none of the genes were found in the second metagenomes of those patients, indicating that the genetic determinant of resistance was acquired at the time of sampling and was not detectable before or after the positive culture of the isolate (Fig. 2A). We did not observe any impact on the acquisition of culturable MDROs or the amount of AMR genes (Resistome: p-value = 0.7787) (Fig. 2B), but we observed a decrease in the amount of typable plasmid at timepoint T2 (Inc-type: p-value = 0.0089)(Fig. 2C). We also observed a positive correlation between the resistome and the amount of typable plasmid, indicating that the majority of the AMR genes were carried on plasmids (Pearson correlation: R²=0.47, p-value < 0.001). We did not observe a significant impact of sex, age or antibiotic usage at the time of sampling or in the last 6 months on the resistome.

### Strain level comparison

**Fig. 3:**
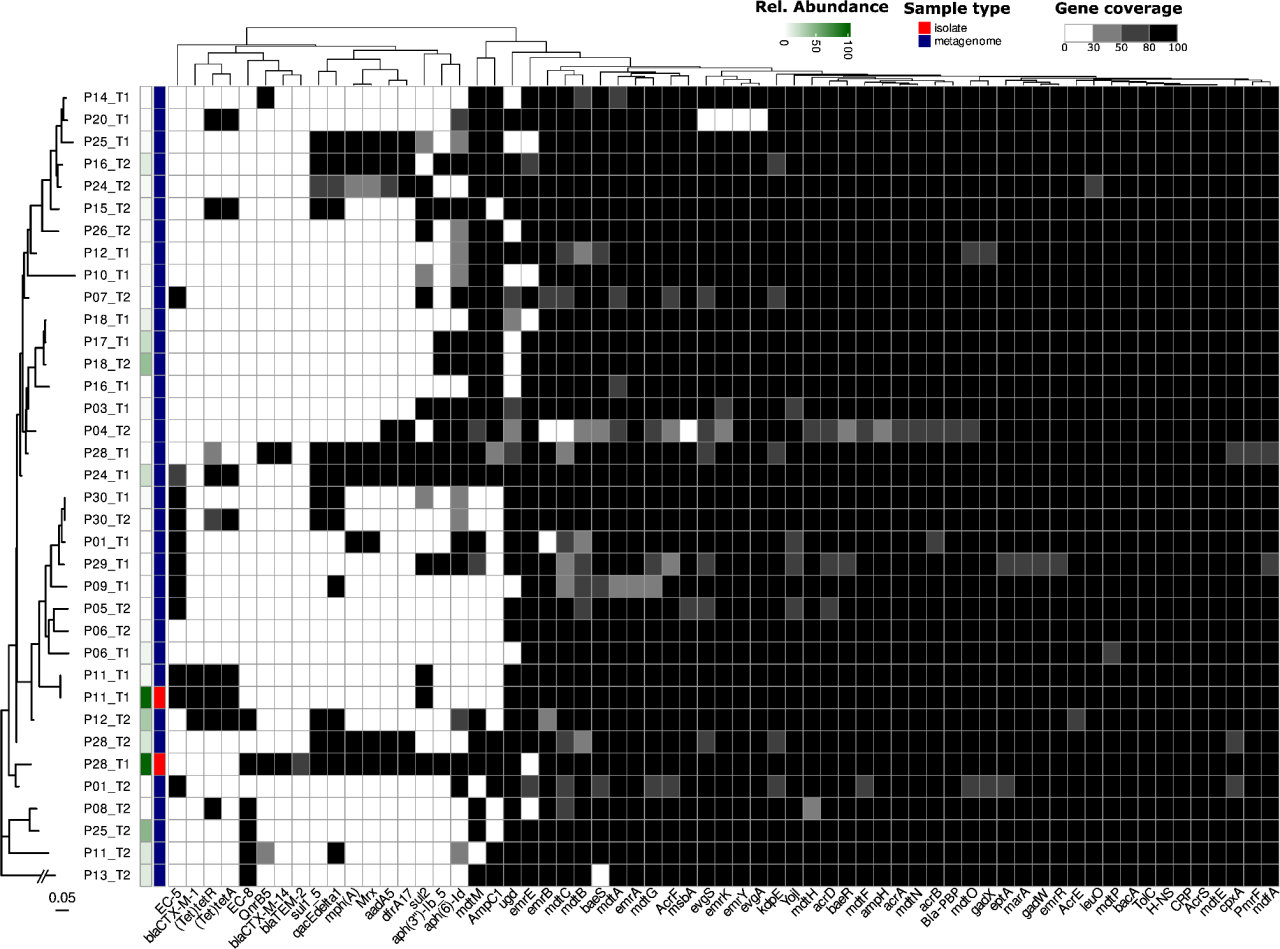
Phylogenetic tree at the strain level using StrainPhlAn of the *E. coli* from our study. The heatmap represents the AMR genes detected in either the genomes from isolated *E. coli* or the metagenomes. The AMR genes displayed were selected based on their presence in at least one of the isolated *E. coli strains*

Of the 52 metagenomes, 45 contained *E. coli*, but only 34 samples had enough markers in the metagenome to perform strain analysis for comparison to the isolated strains. We observed good concordance between the strains isolated from P11 at timepoint 1 and the corresponding strain in the metagenome (0 mutations over 42190 sites), while the strain found at timepoint 2 was more phylogenetically distant (> 1000 mutations). We also observed full concordance of the AMR genes between the isolated strain and the corresponding metagenome (Rel. abundance: 4.2%). For Patient 28, we observed a different pattern in which the isolated strain was closer to timepoint 2 (73 mutations/41702 sites) than to timepoint 1 (144 mutations/41230 sites) when it was originally isolated. We also observed good concordance between the antimicrobial resistance genes of the isolated strains and the corresponding metagenome (Rel. abundance: 1.3%), where only two genes (*bla*_EC−8_ and *bla*_TEM−2_) were missing from the metagenome. Only one patient had closely related *E. coli* strains (P30: 2/41601 mutations) between the time points. These two strains are the only ones closely related to a level corresponding to the previously published mutation rate of 6.5*10^-7 [25]. We also detected closely related strains between P18 and P17 at timepoint 1 (11/42972 mutations) (Fig. 3).

**Fig. 4:**
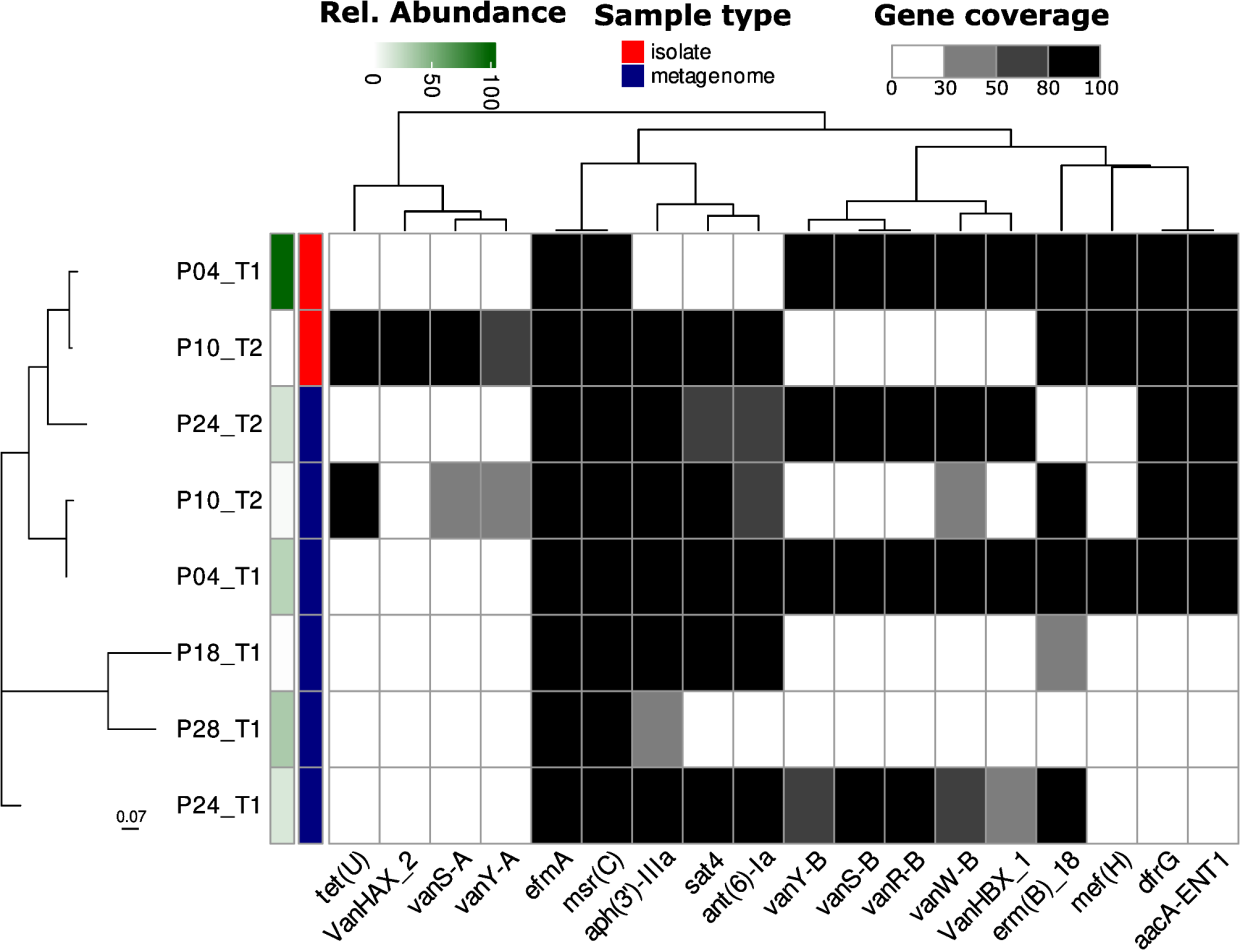
Phylogenetic tree at the strain level using StrainPhlAn of *E. faecium* from our study. The heatmap represents the AMR genes detected in either the genomes from isolated *E. coli* or the metagenomes. The AMR genes displayed were selected based on their presence in at least one of the isolated *E. faecium strains*

We found *E. faecium* in 12 metagenomes with a relative abundance ranging from 0.001 to 32.9%, but we never found *E. faecium* in two consecutive timepoints within a patient except for P24. We observed a close genetic relationship between the P4-T1 and P10-T2 strains from the metagenome (11/256599), with a mutation rate of 4.3 * 10 − 5, which is related to the 25 published mutations per year per genome [26]; surprisingly, the strains from the metagenome to their isolated counterparts were phylogenetically distant (P4: 126/262138; P10: 716/256987). We identified the *van* cassette in every metagenome where a VRE was isolated as well as in samples P24-T1 and P24-T2, where the relative abundance of *E. faecium* was relatively high (13.9% and 16.9%, respectively) (Fig. 4). No growth of vancomycin-resistant organisms was observed at T1, but we did observe the growth of *E. gallinarum* on selective agar at T2. The relative abundance of *E. gallinarum* in the metagenome was 0% at time T1 and 0.008% at time T2.

We detected *K. aerogenes* in 4 metagenomes, but the relative abundance was less than 0.1% in all the metagenomes except P20-T2 (11.1%), which is the sample with a positive culture. Due to the low prevalence, we could not perform a strain analysis. However, we observed good concordance between the AMR profile of the P20-T2 metagenome and that of the isolated strain, and P20-T1 did not contain *K. aerogenes* (Suppl. Figure 3A). We observed the same pattern as for *K. pneumoniae*; only 8 metagenomes were positive (rel abundance ranging from 0.006 to 3.3%), and the sample with a culture positive for MDR *K. pneumoniae* P24-T2 had one of the highest abundances (1.0%), but we did not find *K. pneumoniae* in P24-T1. The AMR profiles of the isolates from P24-T2 were fully covered within the metagenome P24-T2 (Suppl. Figure 3B).

## Discussion

In a proof-of-principle study, we showed that mNGS of rectal swabs can detect clinically relevant AMR profiles. However, our data also indicate that the rectal microbiome is variable, with high β diversity between sampling timepoints. Consequently, the presence of pathogenic species and ARG profiles do not correlate well with classical culture-based diagnostics. Additional studies are warranted to investigate whether such discrepancies are due to transient or niche colonization of MDROs, detection limits of both culture-based and sequencing-based methods, or variability of the sampling and specimens.

The acquisition of nosocomial MDRO colonization and infection in high-risk patients is often associated with high mortality, so infection prevention and control measures are often enforced for these patient populations [27, 28]. A key to successful preventive containment is the timely identification of asymptomatic MDRO carriers. However, the identification of asymptomatic carriers can be challenging due to the sensitivity of the implemented screening algorithms [29]. Although classical diagnostics using selective culture and targeted molecular approaches are highly valuable, the utility of clinical metagenomics is still controversial.

Our results indicate that rectal swabs can be used for microbiome analysis. We observed high temporal variability between the time points, indicating that the microbiome of the rectum is fluctuating but with strong co-occurrence of taxa within the population, suggesting that it is a regulated niche with a cooperative biotic interaction between multiple taxa [30]. Therefore, rectal swabs cannot fully represent the gut microbiome because they are more variable and represent a different structure from that in the gut. While *Bacteroides*, *Blautia*, *Faecalibacterium* and *Ruminococcus* are the most prevalent and abundant genera in the stool, we observed a more pronounced dominance of the genera *Finelgodia magna*, *Enterococcus species* and *Escherichia coli*, confirming results observed in a comparative study led by Sun et al. [31] but contrasting studies performed on 16 S metagenomic [32] or mNGS [8], which revealed a good association at the taxonomic level and concluded that rectal swabs are a good surrogate for gut microbiome studies. One of the reasons for this difference could be the sampling method, which depends on the bacterial density, fecal matter being visible and how the swab is taken [33]. Therefore, sampling quality and strategy are key in conducting studies aiming to investigate the gut microbiome. In a study performed by Boid et al. [34], only swabs with visible fecal matter were included, and their results showed that ESBL acquisition did not affect the diversity or overall structure of the microbiome, which is consistent with our results in which we did not observe a major impact of MDRO acquisition or loss on the microbiome.

Conventional microbiological culture for MDRO detection is easy to perform and is often used to study the dynamics of longitudinal MDRO carriage [35]. However, culture-based MDRO detection methods or targeted molecular methods may not provide a complete picture of the microbiome and resistome and therefore may not allow for differentiation between the de novo acquisition of MDROs or whether MDRO detection is facilitated by selection pressures such as antibiotic exposure. Our results showed that the AMR profiles of the isolated MDROs were almost completely recovered in the rectal swab metagenome, despite a low relative abundance (< 5%) of the bacterial species in the metagenome in 4/6 patients. This indicates the good sensitivity of the method for detecting AMR genes, although AMR detection was performed on assembled data, which is therefore less sensitive than detection from the raw reads but offers a lower false positive detection rate [36]. In the three patients with isolated MDROs at time T2, we could not detect ARGs in the T1 samples, indicating that the ARGs and MDROs were acquired at the time of the sampling and did not originate from horizontal gene transfer of the ARGs between the commensals and the pathogenic bacteria. In the patients with MDRO carriage at time T1, we also did not observe long-lasting colonization, as described in other studies, indicating that the strains no longer colonized the rectum, while our time scale was equivalent (1–2 years interval) [2]. These conclusions are strengthened by the fact that in all cases with *E. faecium* and *Klebsiella* species, the species of interest were absent from the metagenome before/after the time point. Only patients with *E. coli* showed consecutive detection, most likely due to the prevalence and stability of the species in the gut microbiome [37]. However, strain analysis of *E. coli* revealed a limited phylogenetic relationship between consecutive metagenomes, suggesting that strain replacement or at least strain diversity occurred in the gut microbiome. Due to this diversity, the most abundant strain at the time would be the strain we are looking at the moment, therefore blurring the phylogeny [38]. It is logical that we observed a quite volatile resistome between the two time points, especially regarding MGE and beta-lactamase genes, due to the temporal variation in the microbiome itself.

We detected a greater abundance of ARGs than in other studies, and the number of ARGs detected correlated more strongly with an older population, most likely due to the mean age of our cohort (62 years) [10, 39]. Most of the ARGs are chromosomally encoded and are linked to the prevalence of their host. These ARGs are less likely be disseminated through the microbiome and, therefore, would not pose a problem in spreading to other species and pathogens [40]. However, we also detected a significant association between the number of plasmids (Inc-type) and the resistome, indicating a clear impact of MGE on the resistome. Furthermore, we detected beta-lactamases in every metagenome of this study. The most prevalent beta-lactamases are associated with *Bacteroides*, such as *cepA* or *cfx*-like genes, which is an interesting finding as it has been shown that these genes, as well as the genus *Bacteroides*, are less represented in rectal swabs than in the stool/gut microbiome [8]. We also detected a low prevalence of *Bacteroides*, but this did not seem to hamper the detection of the ARGs when we compared our prevalence with published previous work [8, 41]. The prevalence of transferable beta-lactamase genes such as *bla*_TEM_, *bla*_SHV_ and *bla*_CTX−M_ is also of concern because, although we did not grow MDROs from rectal swabs, this indicates that MDROs are present in the samples but most likely are not carried by the targeted organisms (*Enterobacteriaceae* and *Enterococci*) but rather are commensals, suggesting that commensals may be important reservoirs for ARGs in the microbiome. However, these ARGs may be transferred between genera and can, under antibiotic pressure, promote the selection of pathogenic MDROs [42, 43]. The presence of beta-lactamase genes in commensals and bystanders may translate to reduced efficacy of beta-lactam antibiotics through community protection from the commensal carrier [44–46]. Similarly, the prevalence of the *van* cassette and *mec* cassette in samples where *E. faecium* and *S. aureus* are present does not necessarily translate to the presence of VRE, methicillin-resistant *S. aureus* (MRSA) or vancomycin-resistant *S. aureus* (VRSA). These resistant determinants are often found in commensals, and therefore, inferring the presence of MDROs from the co-occurrence of ARGs and pathogenic species in the same samples is flawed and should always be done with caution. In the majority of patients in our study, we detected beta-lactamase genes or *van* cassettes without culturable MDR Enterobacteriaceae or *E. faecium*. Based on these findings and limitations in the interpretation, ARG detection alone from mNGS should not be used to guide antimicrobial therapy [47].

Overall, our results indicate that rectal swabs can be used for AMR screening and molecular diagnostics and that rectal swabs are valuable samples for longitudinal studies because they also allow profiling of the gut microbiome in patients who cannot produce stool at the time of sampling; however, these methods also have some limitations. First, the number of patients who could be included was limited because it was quite rare to find two consecutive rectal swabs from the same patient within the four-year follow-up period, and the initial study was not designed as a follow-up study. We also observed a limited number of MDROs with only 6 positive samples, but this result is due to the low prevalence of MDROs in our clinical setting. Because we did not have paired matched stool samples, we cannot evaluate whether the swabs represented real carriage within the gut microbiome, and some patients without MDRO carriage might have been carriers within the gut/stool microbiome (i.e., false negatives). Finally, we did not proceed in parallel sterile swabs to evaluate the impact of the handling and storage on potential contamination. The samples were handled for microbiological diagnostic by culture before being frozen and stored which increase the risk of contamination. While all these limitations potentially affect the generalizability of the impact of MDROs on the microbiome and the temporal stability of the rectal swab microbiome, they do not affect the ability of mNGS to detect resistance genes at the time of positivity or several potentially transferable ARGs in samples without the growth of pathogenic MDROs.

Our study showed that the rectal swab metagenome can correctly reflect the ARG and AMR profiles of MDROs contained within the microbiota, even if the relative abundance is low. We also showed that the prevalence of beta-lactamase and vancomycin resistance cassettes is quite high and co-occurs with the abundance of Enterobacteriaceae and Enterococci, respectively. Therefore, our study suggested that mNGS can complement microbiological diagnosis, but the inference of a carriage with an MDRO based solely on the co-occurrence of the resistant determinant and the classical bacterial hosts should not be the only criterion for interpretation and should be coupled with a secondary validation method, such as conventional culture.

## Electronic supplementary material

Below is the link to the electronic supplementary material.

Supplementary Material 1

## Data Availability

Short-read data (fastq files) were submitted to the Sequence Read Archive (SRA) under the Bioproject PRJNA1118222 and the accession number (ERR3618849 & ERR3434113).
