# Supplementary material for: Towards unraveling antimicrobial resistance dynamics: a longitudinal exploration of rectal swab metagenomes
Source: BMC Microbiol. 2025 Mar 17;25:150. doi: 10.1186/s12866-025-03874-z (PMC11912604; doi:10.1186/s12866-025-03874-z)

**Title:**

***Towards unravelling antimicrobial resistance dynamics: A longitudinal exploration of rectal swab metagenomes*.**

**Supplementary figure 1.** Co-occurrence network based on Spearman correlation between the relative abundance of each species. Only correlation with an absolute Rho ≥ 0.5 and a p-value ≤ 0.01 are displayed.


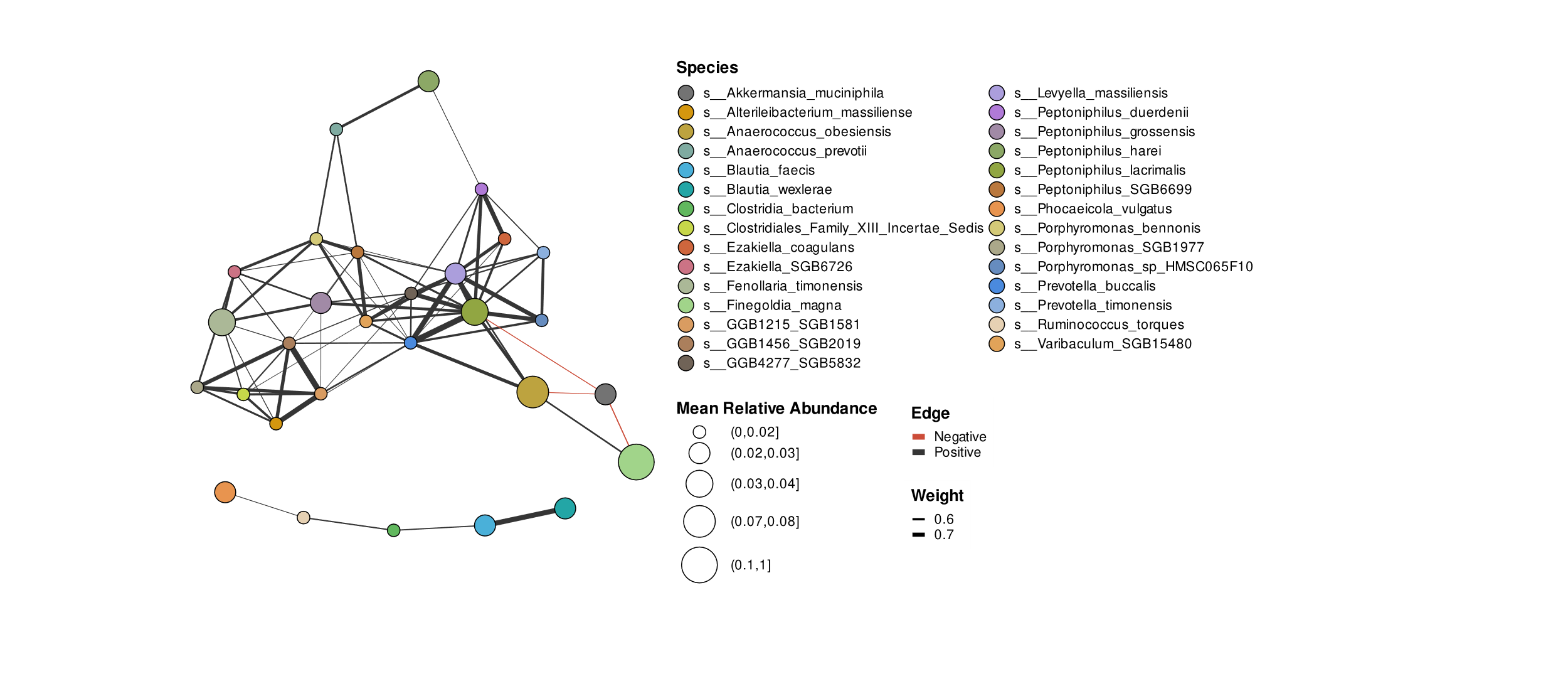


**Supplementary figure 2**. Impact of the timespan between the time points on the β-diversity measured by the Morisita-Horn dissimilarity index.


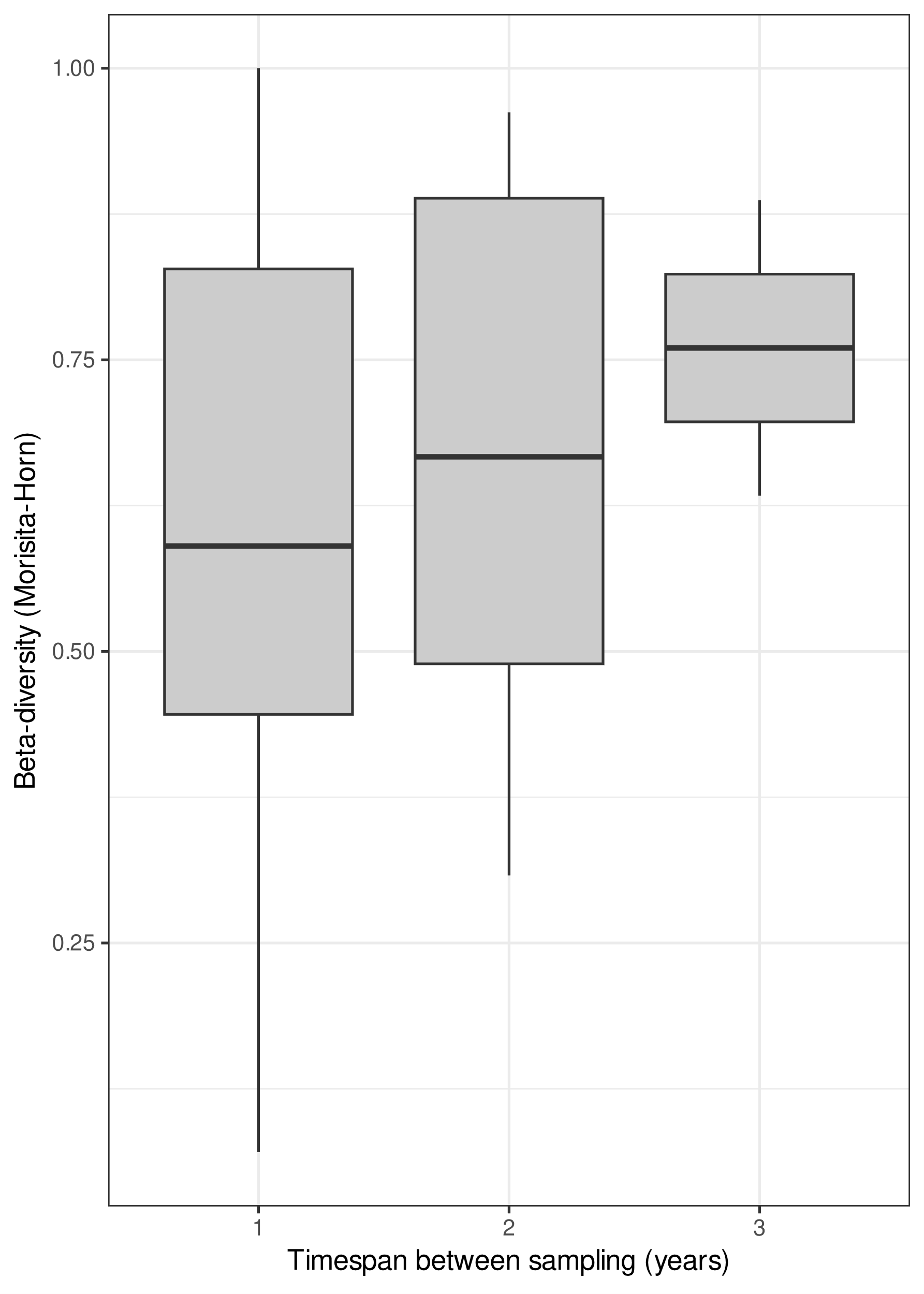


**Supplementary figure 3**. Heatmap represents the AMR genes detected in either the genomes from isolated *K.aerogenes* (A) or *K. pneumoniae* (B) and their corresponding metagenomes. The AMR genes displayed were selected based on their presence in at least one of the isolated strains.


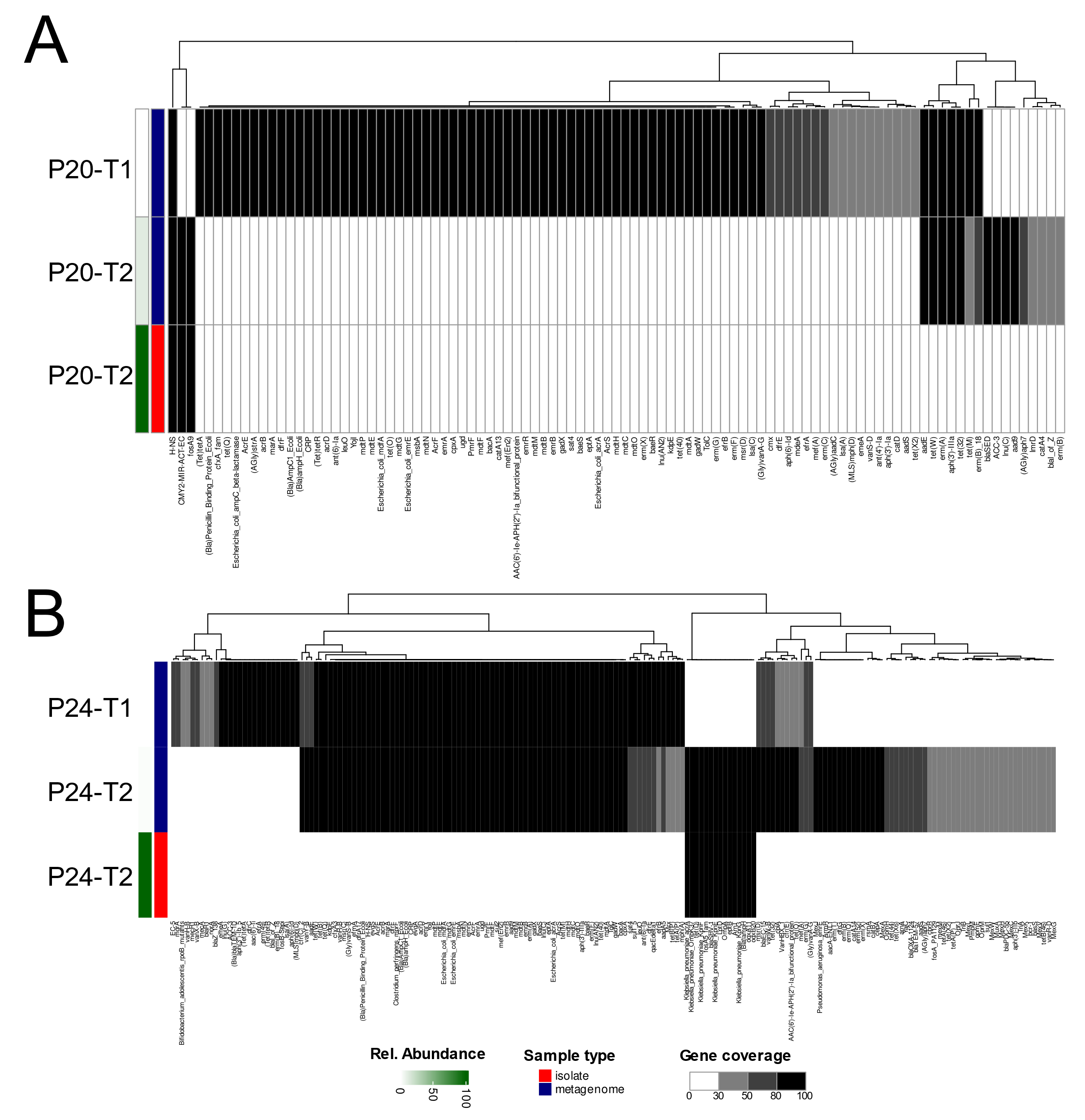

Supplement: Supplementary file 1 — Supplementary Material 1 [file 12866_2025_3874_MOESM1_ESM.docx]
